# From the animal house to the field: Are there consistent individual differences in immunological profile in wild populations of field voles (*Microtus agrestis*)?

**DOI:** 10.1371/journal.pone.0183450

**Published:** 2017-08-17

**Authors:** Elena Arriero, Klara M. Wanelik, Richard J. Birtles, Janette E. Bradley, Joseph A. Jackson, Steve Paterson, Mike Begon

**Affiliations:** 1 School of Life Sciences, University of Nottingham, Nottingham, United Kingdom; 2 Department of Zoology and Physical Anthropology, University Complutense of Madrid, Madrid, Spain; 3 Institute of Integrative Biology, University of Liverpool, Liverpool, United Kingdom; 4 School of Environment and Life Sciences, University of Salford, Salford, United Kingdom; Imperial College Faculty of Medicine, UNITED KINGDOM

## Abstract

Inbred mouse strains, living in simple laboratory environments far removed from nature, have been shown to vary consistently in their immune response. However, wildlife populations are typically outbreeding and face a multiplicity of challenges, parasitological and otherwise. In this study we seek evidence of consistent difference in immunological profile amongst individuals in the wild. We apply a novel method in this context, using longitudinal (repeated capture) data from natural populations of field voles, *Microtus agrestis*, on a range of life history and infection metrics, and on gene expression levels. We focus on three immune genes, IFN-γ, Gata3, and IL-10, representing respectively the Th1, Th2 and regulatory elements of the immune response. Our results show that there was clear evidence of consistent differences between individuals in their typical level of expression of at least one immune gene, and at most all three immune genes, after other measured sources of variation had been taken into account. Furthermore, individuals that responded to changing circumstances by increasing expression levels of Gata3 had a correlated increase in expression levels of IFN-γ. Our work stresses the importance of acknowledging immunological variation amongst individuals in studies of parasitological and infectious disease risk in wildlife populations.

## Introduction

It is well known that inbred strains of mice, living in simple environments far removed from nature, differ immunologically, with implications for the way in which they respond to infection [1–4]. However, little is known about whether these patterns extend to natural populations, and indeed to differences between individuals within natural populations, which are typically outbred and face a multiplicity of challenges, parasitological and otherwise. Such individual differences may in turn affect how natural populations respond to infection, with potentially important consequences for the conservation and control of wildlife.

Work carried out on natural populations has focused largely on a select few measures of immunity, for example, heterophil/lymphocyte ratio or IgG level [5,6], and has typically considered immunity as a single trait of an individual, which can be misleading [7]. Immunity, rather, comprises multiple, interacting traits; for example the different types of response co-ordinated by T-helper cell (Th) phenotypes. We examine a subset of these here to provide a multivariate representation of the immune system; and, furthermore, one that maps more tractably onto concepts of immune regulation derived from laboratory immunology [8]. We seek any significant relationships between such traits. But we focus in particular on identifying consistent differences in immunological profile, here defined as the expression profile of a selection of immune genes, between individuals within natural populations. This builds on our previous work, which shows evidence for differences in immune strategy between males of different ages within the same populations [9].

In practical terms, consistent differences between individuals may be said to exist if levels of expression of a character are detectable from multiple observations of those individuals, after various factors known to affect that character, and experienced by the individuals, have been taken into account. Inevitably, the set of factors taken into account can never be exhaustive. The consistent differences observed may result from an individual's genotype (often, as here, unknown) or other, unaccounted-for factors. These unaccounted-for factors can be divided into two categories. First are those experienced early in the individual’s life, before the first observation, or acting consistently on individuals throughout the series of observations (e.g. a chronic infection). These may contribute to the consistent differences observed. Second are those acting inconsistently across the series of observations, just like those factors that are accounted for. Failing to account for these will tend to hide consistent differences. The respective contributions of genotype and past environment to consistent individual differences are important to understand, but that is not the issue we address here. Regardless of cause, it is important to first determine whether such differences exist and can be detected within natural populations and the nature of such differences, as this could have important implications for our understanding of parasitological and infectious disease risk in the wild.

At the within-individual level, one key immunological association extensively discussed in the context of co-infection is that between the Th responses [10]. Activated Th cells adopt differentiated phenotypes that promote functionally distinct immune responses [11], helping the body tailor defences to the range of pathogenic challenges it may face. A particular dichotomy (very well studied in the laboratory) is found between the Th cell type 1 (Th1) and Th cell type 2 (Th2) phenotypes [12], which respectively promote defences effective against intracellular microparasites (e.g. viruses, intracellular bacteria) and macroparasites (e.g. helminths). Many pathogens trigger mixed Th1 and Th2 commitments [13], and their degree of functional independence may depend on the context. Nevertheless they are antagonistic via their (mutually inhibitory) key molecular signals [11] and represent distinctive phenotypic commitments that could compete for resources.

In the present study, we focus on these well understood Th phenotypes as markers of distinct immune commitments and also consider immunosuppressive, regulatory immune responses of a type known to limit effector activity and the risk of immunopathology. We measured mRNA expression of the genes interferon gamma (IFN-γ), a pro-inflammatory cytokine important in Th1 responses, GATA binding protein 3 (Gata3), a master transcription factor involved in the development of Th2 responses, and IL-10, an immunosuppressive cytokine produced by many immune cell types including regulatory Th cells (Treg). Although we only use one gene to represent each phenotypic commitment, the functional significance of their measured expression levels in immunological cell populations from wild field voles is supported by our previous studies. For IFN-γ and IL-10 expression, multivariate patterns of co-expression with functionally related genes [9] suggest they may be interpreted as part of wider Th1 and Treg responses (respectively). For Gata3 expression, we have found epidemiological association with macroparasite infection [14], consistent with its putative role in Th2 responses.

The populations used here, of field voles, *Microtus agrestis*, from Kielder forest, UK, have previously been the subject of a series of parasitological and eco-immunological studies (reviewed in [15], where more detailed references may also be found). Briefly, the voles inhabit grassy clear-cut areas where trees have been felled in what is a managed coniferous forest. They exhibit a pattern of multi-annual fluctuations in abundance with peak vole densities occurring at 3–4 year intervals, followed by steep population collapses usually taking place in summer during the vole breeding season, and followed by up to a year with little noticeable population growth. Within a single grass patch, peak densities span 5–770 voles ha^−1^. The voles have life histories typical of microtine rodents, with high fecundity (an average litter size of five), a low age at maturation for some seasonal cohorts (as little as 28 days old for spring and early summer-born females), and birth intervals by members of overlapping cohorts as short as 21 days during a breeding season. However, for individuals born in the second half of the summer, reproduction is typically delayed until the next spring. Juveniles and subadults, but also females breeding in the year of birth, have non-defended home ranges, though overwintered females do tend to defend territories. Dispersal is primarily by subadults. Infection dynamics in the voles have been studied most extensively for a number of microparasite species, notably cowpox virus, *Bartonella* spp., *Mycobacterium microti* (vole tuberculosis) and *Trypanosoma microti*, but also for fleas, ticks and helminths.

Arguably, wild rodents provide a unique link between the biomedical knowledge gained from laboratory rodents and the genetic, ontogenetic and environmental variation observed in human and other natural populations [16]. Their short lifespan, coupled with our monthly sampling regime, also allows us to search for long-term consistensies, across the entire lifespan of an individual. Previous studies have stressed the importance of such large time scales (relative to the life span of the study species), which span “ecological and evolutionary scales of time and spaces”, in the context of behavioural consistencies or ‘personalities’ [17].

Given the larger amount of variation expected within a wild population, as opposed to laboratory stocks, sufficient numbers of repeated measures are critical in teasing apart within- and between-individual effects. However, very few studies have measured multiple immune markers in a wild animal over a series of time points (but see [6,18]). Here, therefore, we explore the expression of markers for Th1, Th2 and regulatory activity using a large longitudinal dataset (with multiple repeated measures from the same individual) from wild populations of field voles, *M*. *agrestis*. To our knowledge, this is the first study in which gene expression data have been used to search for consistent individual immunological differences.

## Materials and methods

We studied *M*. *agrestis* in Kielder Forest, UK (55°13' N, 2°3' W) using live-trapping to access individual animals from natural populations. Access to the study site was provided by the Forestry Commission. Our study was designed to permit the analysis of individual variation in condition and survival, infection status, and the expression of immune genes (for full details of all methods below see [9,14]). Briefly, we repeated our field design at two spatially separate sites in 2008–2009, and a further two in 2009–2010. Each site was monitored by monthly trapping sessions between February (2008–2009) or April (2009–2010) and November, and contained a live-trapping grid (~0.375 ha) of 150 (10x15) regularly spaced traps (3–5 m intervals) placed in optimal habitat. Animals were marked with passive radio frequency transponders (AVID) and monitored over time, as sequences of capture and recaptures. At each capture, biometric, infection, and immune expression measurements were taken. All animal procedures were performed with approval from the University of Liverpool Animal Welfare Committee and under a UK Home Office license (PPL 40/3235 to MB). All efforts were made to minimise suffering.

Gene expression levels of IFN-γ, Gata3, and IL-10 were measured by Q-PCR from peripheral blood samples. Ectoparasite infections were recorded as direct counts or semi-quantitative abundance indices (from 0 = absence to 4 = high density) of myobiid fur mites, listrophorid fur mites, laelapid mites, ticks (*Ixodes* spp.), lice (*Hoplopleura acanthopus*), small flea species (*Ctenophthalmus nobilis*, *Peromyscopsylla spectabilis*, *Megabothris walkeri*, *Malaraeus penicilliger*, *Rhadinopsylla pentacantha*), or large, slow-moving fleas (*Hystrichopsylla talpae talpae)*. Infectious status by microparasites (*Babesia microti*, *Bartonella* spp., cowpox virus) was determined using previously established PCR protocols [19,20]. For infection statistics for the parasites measured in this study see [9].

### Statistical methods

Our overall strategy was to seek consistent differences between individuals either in single traits, or in combinations of traits, after variations in those traits attributable to other factors that we monitored had been taken into account. Individual consistency can only be detected if those individuals are sampled multiple times. Hence, we used a dataset consisting of 248 measurements of immune gene expression levels in the 60 individuals sampled between 2–7 times, and over a mean time period of 102 days (range = 55–169). Given that the median survival rate of Kielder field voles has been estimated as 0.63 per lunar month [21], such that *c*50% of voles are likely to die in 44 days, *c*75% in 88 days and *c*90% in 132 days, we were able to capture the majority of a vole’s life.

We fitted a multivariate mixed-effects model in a Bayesian framework using Markov chain Monte Carlo (MCMC) methods in the package MCMCglmm [22], in R 3.2.0 [23]. Multivariate mixed-effects models are increasingly used in studies distinguishing between within-individual and between-individual correlations between behavioural traits (see [24] for a recent discussion) and have recently been applied to eco-immunology [25]. In our analyses, the three immune genes were log-transformed, standardized and included as dependent variables with Gaussian distributions.

We used an inverse gamma prior, an MCMC sampling scheme of 13000 total iterations with a 3000 iteration burn-in and thinning interval of 10 [22]. To ensure convergence, we combined visual checks with a Gelman-Rubin diagnostic [26]. Posterior modes or means along with their 95% credible intervals (95% CRI; based on the 95% Highest Posterior Density) are presented. As in previous studies, we report whether the 95% CRI for our repeatability and covariance estimates include zero, as an indication of statistical significance (as in e.g. [27,28]). However, we also performed a second checking step which involved running the same multivariate mixed-effects model on 1000 random permutations of the original dataset (without replacement) to generate *P* values for these estimates.

Prior to fitting our multivariate mixed-effects model, we fitted univariate mixed-effects models with each of the three immune genes as dependent variables in separate models, to determine the best set of predictors explaining variation in the expression levels of each of these. This was done by constructing global models including all predictor variables of interest, and subsequently simplifying these using a stepwise approach based on AIC and likelihood ratio tests. The full list of predictor variables included was: trapping session, year, site of capture, sex, morphological measurements (including body condition), reproductive stage, infection with cowpox virus, *Bartonella* and *Babesia*, and an index of ectoparasite infestation (as in [14], to reduce the number of predictor variables). The predictors that best explained variation in expression levels of IFN-γ, Gata3, and IL-10 were infection with cowpox virus, *Bartonella* and *Babesia* (reference category for each of these set to uninfected), sex, site of capture and body condition. Hence, these were included as fixed effects in the multivariate model. A random effect of individual ID was included in all univariate and multivariate models.

## Results

### Predictors of variation in expression levels of immune genes

Higher expression levels of IL-10 and IFN-γ were associated with current infections with *Babesia* and *Bartonella*, and cowpox virus, respectively, while lower expression levels of Gata3 were associated with cowpox virus infection and marginally associated with *Babesia* and *Bartonella* infection (Table 1). In addition, expression levels of Gata3 were lower in males than in females, and differed between certain trapping sites (Table 1).

**Table 1:** Results of multivariate mixed-effects model with standardized values for the three immune effectors.

	Posterior mean	95% credible interval	*P* value
**IL-10**			
Body condition	-0.032	(-0.104, 0.046)	0.406
Site (SCP)	0.067	(-0.485, 0.689)	0.808
Sex (M)	0.249	(-0.113, 0.610)	0.162
Cowpox	0.347	(-0.134, 0.862)	0.190
Babesia	**0.464**	**(0.162, 0.816)**	**0.002**
Bartonella	**0.479**	**(0.197, 0.714)**	**<0.001**
**GATA3**			
Body condition	-0.003	(-0.073, 0.073)	0.958
Site (SCP)	**-0.955**	**(0.385, 1.543)**	**0.002**
Sex (M)	**-0.525**	**(-0.903, -0.213)**	**0.004**
Cowpox	**-0.569**	**(-1.135, -0.068)**	**0.042**
Babesia	-0.297	(-0.593, 0.010)	0.056
Bartonella	-0.222	(-0.458, -0.042)	0.088
**IFN-γ**			
Body condition	0.007	(-0.070, 0.082)	0.840
Site (SCP)	0.197	(-0.399, 0.821)	0.516
Sex (M)	-0.159	(-0.508, 0.277)	0.408
Cowpox	**0.734**	**(0.293, 1.274)**	**0.008**
Babesia	0.250	(-0.049, 0.575)	0.106
Bartonella	0.143	(-0.164, 0.393)	0.270

Significant effects shown in bold type.

### Consistent individual differences in expression levels of immune genes

*M*. *agrestis* appeared to show consistent individual differences in their expression levels of at least one of the immune genes measured, but there was some evidence for such consistent differences in all three. For all immune genes, the 95% CRI did not include zero (Table 2), but the significance values generated from random permutation tests suggested that only IFN-γ expression was significantly repeatable (IFN-γ: *P* = 0.01; IL-10: *P* = 0.12; Gata3: *P* = 0.37).

**Table 2:** Repeatabilities of gene expression levels for IL-10, Gata3 and IFN-γ with 95% credible intervals (CRI) extracted from the multivariate mixed-effects model.

Trait	Repeatability	Lower 95% CRI	Upper 95% CRI
**IL-10**	0.152	0.072	0.242
**GATA3**	0.129	0.069	0.233
**IFN-γ**	0.200	0.106	0.340

### Decomposing correlations between expression levels of immune genes

The correlation matrices for the multivariate mixed-effects model revealed significant residual covariance between expression levels of Gata3 and IFN-γ, That is, there was a significantly positive within-individual correlation between these two genes (Table 3; 95% CRI: 0.290, 0.539; *P* < 0.001). Neither of the other two within-individual correlations were significant (95% CRI: -0.065, 0.164; 95% CRI: -0.220, 0.014). There were no significant between-individual correlations between any of the genes.

**Table 3:** Correlation matrices between expression level of IFN-γ, Gata3 and IL-10 at the between-individual and within-individual level from the multivariate mixed-effects model.

	IL-10	GATA3	IFN-γ
**Between-individual correlations**			
**IL-10**	-		
**GATA3**	0.013 (-0.058, 0.087)	-	
**IFN-γ**	0.003 (-0.090, 0.093)	0.038 (-0.051, 0.141)	-
**Within-individual correlations**			
**IL-10**	-		
**GATA3**	-0.093 (-0.220, 0.014)	-	
**IFN-γ**	0.057 (-0.065, 0.164)	**0.421 (0.290, 0.539)**	-

Posterior means and 95% CRI are shown (95% CRI not including zero are shown in bold type).

## Discussion

We sought and found evidence for consistent individual differences in immunological profiles in natural populations of *M*. *agrestis*.

Our analyses showed variations in expression associated with current infections. These included higher expression levels of IFN-γ associated with cowpox virus infection, consistent with an up-regulated Th1 response to viral infections [29]. Similarly, IFN-γ is known to contribute towards resistance to poxviruses, but immunomodulatory effects of poxviruses on IFN-γ have also been described [30–33]. In addition, lower expression levels of Gata3 were associated with cowpox virus infection. This is consistent with reciprocal regulation of Th1 and Th2 responses [11,12], of which IFN-γ and Gata3 are mediators respectively. It is also opposite to the response identified to macroparasites (helminths and arthropods) and to vole tuberculosis (*Mycobacterium microti* infection) in this system [14], for which Gata3 has been identified as a biomarker of tolerance in mature male voles. However, previous work has shown that deficiency in Th2 cytokine responses can exacerbate orthopoxvirus infection, suggesting that susceptibility/resistance to such viruses may not be as simple as a balance between Th1 and Th2 responses [34].

Most pertinently here, though, our results suggest consistent individual differences in immunological profile within our field vole populations. There was clear evidence of consistent differences between individuals in their typical level of expression of at least one gene, and at most all three genes, after other sources of variation in expression levels had been taken into account. We acknowledge that these variations may themselves reflect differences between individuals that are either genetic or in the levels and effects of other, unmeasured elements of the individuals’ past or consistent present environment. Unlike the controlled environent of the animal house, it is impossible to account for all of the factors acting on an individual in the wild. For example, athough we were able to measure a range of micro- and ectoparasites as part of this longitudinal study, we were unable to estimate the worm burden of individuals; which can be associated with chronic infection [35] and increased expression of Gata3 [14]. It is worth noting though, regarding this particular example, that our data provide the strongest evidence for consistency in IFN-γ, an immune gene associated with the Th1, and not the Th2 response.

The repeatabilities we present are comparable in size to repeatabilities quoted for other immune parameters, albeit in different contexts [6,18]. Our work also highlights the importance of random permutation tests, not widely used previously, for rigorously assessing the significance of repeatability estimates when dealing with such traits and using data from natural populations within a Bayesian framework.

The absence of any significant between-individual correlations indicates that there was no consistent tendency for individuals with a typically high level of expression of one trait having a consistently high (or low) level for other traits. However, a within-individual correlation means that individuals responding to the changing circumstances in their life by a modification to the expression of one trait have a correlated modification to the expression of a second trait. In the present case, any tendency for individuals to increase their expression levels of Gata3 (an element of the Th2 response) was accompanied by a tendency to increase their expression levels of IFN-γ (an element of the Th1 response), and vice versa. There was a positive correlation between the two.

A trade-off between Th1 and Th2 responses has been proposed, with resources invested in one having to be diverted from the other [11,12], though the usefulness of this hypothesis is under debate [10,13]. Our results provide no evidence for this, but they are limited to only one gene per response, and they do not examine what might happen in response to an identifiable parasitological challenge (as we account for these in our statistical model). However, a similar positive correlation, in this case between the Th1-associated and Th2-associated antibodies IgG2a and IgG1, has been shown in malaria-filaria coinfected mice [36]. When positive rather than negative correlations are found between traits that might compete for the same resources, the conventional explanation is that those individuals differ in the total amount of limited resource that they have at their disposal, such that high performance in one trait is accompanied by high performance in the other [37]. It will be instructive to probe further the origin of the positive correlation described here, and whether this correlation has any generality in natural populations.

We have demonstrated that distinct immunological profiles, well established between laboratory strains [1–4] and livestock breeds [38–41], are also demonstrable within wildlife populations. A major advantage of the field vole system that we have utilized here is that it allows us to follow the same individual over a substantial amount of its lifetime, and hence to identify long-term consistencies in immune response. However, individuals also experience major developmental re-organizations within their lifetimes, such as sexual maturation, as well as step changes in their wider environment. Hence, it will be instructive for future research to address the questions: how stable are these immune profiles across developmental thresholds, and how are they shaped by environmental exposure?

High levels of variability in natural populations necessarily present analytical challenges, but they also allow complex interactions between multiple factors to be explored, with results being more readily applicable to other systems. We hope that future studies will continue to explore the phenotypic variance associated with the many elements of the immune system, to further our understanding of consistent differences in individuals' immune responses, or what could be referred to as ‘immune personalities’ in the wild. We believe this knowledge is invaluable for furthering our understanding of disease risk in wildlife systems. Whether the focus is on the conservation of threatened species or the control of species that are pests, the default position is usually that all individuals may be treated as typical, with variation around a norm considered as noise. If, on the contrary, there are consistent individual differences in responses to infection, then, for example, the resilience of the threatened population in the face of extinction risk, or the recalcitrance of a pest population in the face of biocontrol, may depend largely on the individuals most resistant to or tolerant of infection, rather than some statistical norm. Here, as elsewhere, it may be unwise to ignore individual variation.
